# Regulating Apoptosis by Degradation: The N-End Rule-Mediated Regulation of Apoptotic Proteolytic Fragments in Mammalian Cells

**DOI:** 10.3390/ijms19113414

**Published:** 2018-10-31

**Authors:** Mohamed A. Eldeeb, Richard P. Fahlman, Mansoore Esmaili, Mohamed A. Ragheb

**Affiliations:** 1 Department of Chemistry (Biochemistry Division), Faculty of Science, Cairo University, Giza 12613, Egypt; mattia@sci.cu.edu.eg; 2 Department of Neurology and Neurosurgery, Montreal Neurological Institute, McGill University, Montreal, QC H3A 2B4, Canada; 3 Department of Biochemistry, University of Alberta, Edmonton, AB T6G 2H7, Canada; rfahlman@ualberta.ca (R.P.F.); esmaili@ualberta.ca (M.E.); 4 Department of Oncology, University of Alberta, Edmonton, AB T6G 2H7, Canada

**Keywords:** N-end-rule, cell death, protein degradation, apoptosis, N-terminal arginylation, proteases, caspases, proteolysis, ubiquitination, cancer biology

## Abstract

A pivotal hallmark of some cancer cells is the evasion of apoptotic cell death. Importantly, the initiation of apoptosis often results in the activation of caspases, which, in turn, culminates in the generation of proteolytically-activated protein fragments with potentially new or altered roles. Recent investigations have revealed that the activity of a significant number of the protease-generated, activated, pro-apoptotic protein fragments can be curbed via their selective degradation by the N-end rule degradation pathways. Of note, previous work revealed that several proteolytically-generated, pro-apoptotic fragments are unstable in cells, as their destabilizing N-termini target them for proteasomal degradation via the N-end rule degradation pathways. Remarkably, previous studies also showed that the proteolytically-generated anti-apoptotic Lyn kinase protein fragment is targeted for degradation by the UBR1/UBR2 E3 ubiquitin ligases of the N-end rule pathway in chronic myeloid leukemia cells. Crucially, the degradation of cleaved fragment of Lyn by the N-end rule counters imatinib resistance in these cells, implicating a possible linkage between the N-end rule degradation pathway and imatinib resistance. Herein, we highlight recent studies on the role of the N-end rule proteolytic pathways in regulating apoptosis in mammalian cells, and also discuss some possible future directions with respect to apoptotic proteolysis signaling.

## 1. Introduction: An Overview of Programmed Apoptotic Cell Death

In multicellular organisms, cellular homeostasis is maintained through a fine-tuned balance between cell proliferation and cell death [1]. Deregulation of this “life versus death” balance may result in several human diseases and disorders such as cancer, neurodegenerative disorders, and autoimmune diseases [2,3,4,5]. Mounting lines of evidence, over the last two decades, suggest that either hyper-regulation or hypo-regulation of apoptosis (a format of programmed cell death (PCD)) may underlie the previous pathologies [2,3,4,5]. Tellingly, apoptosis itself is often a balance between pro- and anti-apoptotic molecules [6]. Limited proteolytic processing (restricted proteolysis) is at the nexus of crucial regulatory networks controlling apoptotic cell death [7]. Many proteinases such as caspases and calpains cleave ~500 to ~1000 cellular proteins that are crucial for the proper execution of the apoptotic program [8,9,10]. By executing sequence-specific cleavages in many cellular proteins, activated caspases or calpains either abrogate or alter the functions of these proteins [6,11,12,13,14]. Some of the generated protein fragments, such as the cleaved fragments of Bax [14], BID [13], BIM^EL^ [12], and BRCA1 [11], can have deleterious pro-apoptotic effects, whereas other cleaved protein fragments, such as the cleaved fragments of Lyn kinase [15], synphilin-1 [16], P27^kip1^ [17] and RasGAP [18], may have anti-apoptotic functions. Thus, this dichotomy of functions as a result of restricted proteolysis, in addition to the necessity of apoptosis in multicellular eukaryotes, might account for the acquisition and conservation of caspase cleavage sites (and some calpain cleavage sites) in many proteins during evolution [10,19,20]. So, if restricted proteolysis can generate pro-and anti-apoptotic fragments during apoptosis, how do cells regulate and control the output of such proteolytic (caspase/calpain) cascades which regulates the apoptotic execution reactions and hence impacts cellular fate? A possible avenue for regulating the output of such proteolytic cascades (let alone the proportion of pro-and anti-apoptotic fragments produced in each case) is the difference in metabolic stability among different proteolytically-activated protein fragments generated in each case (pro-apoptotic and anti-apoptotic fragments). Consequently, this would result in pro-apoptotic and anti-apoptotic signaling imbalance, and thus, lead to either cell death or cell survival. Given the irreversible nature of limited proteolysis and the presence of feedback loops (either pro- or anti-apoptotic) originating from the generated proteolytic fragments [6,10], the proteolytically-generated neo N-termini on these fragments present a regulatory possibility for of these proteolytic cascades during apoptotic signaling by the selective degradation of specific proteolytic fragments via the N-end rule degradation pathway. As a result, the N-End rule pathway may play a significant role in determining cell fates upon the induction of apoptotic pathways [10,21].

While cell death can occur subsequent to excessive cellular damage or stress, most cell death programs in mammals are seen as taking place in an active manner, as a result of a specific cascade of cellular signaling events [22,23]. Tellingly, the three most widely-known types of cell death are defined, at least in part, by the morphological features of the dying cell: apoptosis (also designated as type I cell death), autophagic cell death (type II cell death), and necrosis (type III cell death) [22,23].

**Figure 1:**
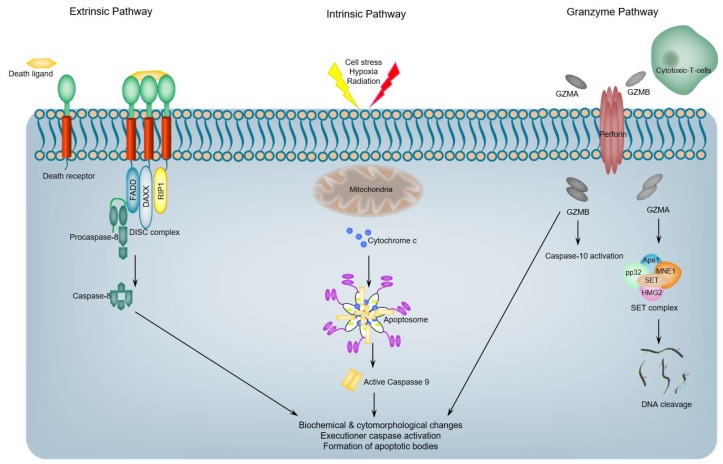
Three apoptotic pathways (Extrinsic, intrinsic and granzyme pathways). In the extrinsic pathway, the extrinsic apoptotic signaling pathway is triggered by ligation of a death receptor, followed by the assembly of a caspase activation platform called the DISC complex. This activation platform recruits and activates caspase-8 via adaptor molecules, and then subsequently leads to caspase 8-mediated activation of downstream effector caspases. In the intrinsic pathway, in response to various cellular stresses, pro-apoptotic proteins of the Bcl2 family mediate mitochondrial outer membrane permeabilization (MOMP), permitting the release of pro-apoptotic factors such as cytochrome c into the cytosol. Cytochrome c then binds to APAF1, mediating its oligomerization and recruitment of caspase 9 into the apoptosome, which in turn leads to the activation of caspase 9 and subsequent proteolytic activation of effector caspases. In the granzyme B pathway, the injection of granzyme B by cytotoxic T-Cells can lead to the direct proteolytic activation of caspase 10, among other targets, which subsequently leads to the activation of effector caspases.

Apoptotic cell death is a inherent form of cellular demise programmed in all normal cells that permits the cell to commit molecular-controlled cellular suicide while avoiding inflammation and damage to neighboring cells and the cell’s environment [22,23,24,25,26]. Tellingly, apoptosis is characterized by distinct biochemical features, including membrane blebbing, cellular shrinkage, chromatin condensation (pyknosis), and DNA fragmentation [26]. It can be further defined as a mode of cell death that is accompanied by caspases (proteases) activation [27]. Apoptosis is generally appreciated to play a crucial role in the survival of multicellular organisms via the elimination and removal of any abnormal or otherwise infected cells that may disrupt the normal physiological functions of the organism [28], although there are diverse apoptotic pathways that can be distinguished by the identity of molecular mediators that include adapters and initiator caspases involved. Apoptotic programs are generally categorized into the extrinsic (death-receptor pathway), intrinsic (mitochondrial) pathway, or granzyme dependent pathway [22,27] (Figure 1). Here, we briefly highlight these three pathways of apoptosis with a major focus on Caspases-mediated, apoptotic signaling, and the regulation of this execution phase. For detailed insights regarding different molecular mechanisms of apoptotic pathways and their extensive mechanisms of regulation, we refer the reader to the comprehensive review by Green and colleagues [22].

The extrinsic apoptotic cell death pathway is initiated via extracellular signals that are transferred to the internal cellular environment via the binding of specific ligands (death ligands) to specific receptors (death receptors (DRs)) [22,27]. Tellingly, death receptors are membrane protein receptors and members of the tumor necrosis factor (TNF) superfamily, and encompass TNF receptor-1 (TNFR1), Fas, death receptor 3 (DR3), DR4 (TRAIL-R1), and DR5 (TRAIL-R2) [22,29]. Death receptor ligands include TNF, CD95-ligand (Fas-L; also, called CD95-L), TRAIL, and TL1A [22,29]. Following the binding of a receptor to its respective ligand, the death receptor transduces a signal to promote the recruitment of the monomeric procaspase-8 protein, via its DED motif, to the death-inducing signaling supra-molecular complex (DISC). This DISC complex is assembled on the cytoplasmic domains of the receptors, such as FAS-associated death domain (FADD) or TNFR-associated death domain (TRADD) [22]. Recruitment of caspase-8 monomers leads to their dimerization and activation, which in turn leads to the proteolytic activation of downstream effector caspases [22,29].

Intrinsic apoptosis is also designated as mitochondrial apoptotic pathway, because it hinges on mitochondrial-derived factors. Importantly, this pathway is activated by a vast spectrum of cellular stress cues, including the withdrawal of a growth factor, partial ablation of cytoskeleton, aggregation of unfolded proteins, and DNA damage. In addition, developmental cues (such as hormones) can also induce apoptotic cell death [30]. At the apex of the proteolytic cascade in the intrinsic pathway is caspase-9, where it has been demonstrated to be the initiator caspase, caspase-9 activation proceeds via a dimerization-dependent mechanism involving interactions with APAF1 [31]. Caspase-9 and APAF1 exist in unstressed cells as inert cytosolic monomers [30,31], but upon the appropriate stress or stimulation, cytochrome *c* is released from the mitochondria. The released cytochrome c subsequently binds the WD domain of APAF1, which results in initiating a cascade of conformational changes that ultimately lead to the assembly of seven of activated APAF1 monomers to form an oligomeric supra-molecular complex, the core of which encompass the CARDs that recruit and activate caspase 9 [32] (Figure 1). The resulting complex apoptosome, which encompasses cytochrome c, caspase 9, and APAF-1, mediates the activation of the caspase 9, which, in turn, can activate downstream effector caspases [32,33].

Since the role of cytochrome *c* in electron transport chain reactions has long been established, it was revealed that mammalian cells devoid of cytochrome *c* are incompetent for caspase activation in response to induction of mitochondrial apoptotic cell death pathway [34]. However, recent work has demonstrated that the role of cytochrome *c* in electron transport is independent from its ability to interact with APAF1 and induce caspase activation and apoptosome formation [35]. Concomitantly, cells derived from knock-in mouse mutant in which residue K72, a crucial residue for APAF1 interaction, of cytochrome *c* was mutated were able to promote electron transport, yet impaired apoptotic cell death [35].

It is imperative to mention that the extrinsic and intrinsic pathways cross-talk via caspase-8 cleavage of the BH3-only protein BH3-interacting domain death agonist (BID), where this cleavage event produces the active, truncated pro-apoptotic form of BID (tBID) that triggers MOMP [13], and subsequently amplify the apoptotic cell death signaling [13,22,23,36].

Activation of effector caspases result in the proteolysis of diverse signaling molecules including other proteases, leading to an amplified proteolytic cascade. It is also noteworthy that the activation of these or related proteases contributes to the activation of calpains which are also activated during apoptotic cell death [22,23,32,35,36]. The overall result may be an escalating cascade of proteolytic processing. Proteolytic cleavage of specific substrates may further contribute to the process of apoptotic cell death through different ways, e.g., via structural changes, by the activation of signaling proteins by the removal of regulatory domains, or by the inactivation of inhibitors [22,23,36].

Among the most characteristic changes associated with apoptotic cell death, chromatin condensation and nuclear changes, and proteolytic processing could play a pivotal role in this context. For instance, lamin B1 degradation during apoptotic progression could lead to collapse of the chromatin due to the severe loss of attachment points on the nuclear matrix [22,23,36]. Other characteristic alterations and changes during apoptotic cell death are related to the plasma membrane and cytoskeleton, as cells lose attachment, undergo blebbing, and fragment. Membrane blebbing and cellular fragmentation into apoptotic bodies depend upon actin polymerization; thus, it seems that the targeted proteolytic cleavages of actin [36] and of the actin-associated protein fodrin are relevant to these cellular alterations regarding the organization of the plasma membrane [22,23,36].

In addition to the extrinsic and intrinsic apoptotic pathways, granzymes, granule-secreted proteases, can mediate apoptotic cell death program through caspase-dependent and -independent molecular pathways [37,38] (Figure 1). In granzyme-mediated apoptotic cell death, granzyme B (GrB) and perforin are released from the granules of cytotoxic T-cells. Granzyme B, which has access to the cytoplasm of target cells by a perforin-mediated endocytosis process, cleaves a number of documented substrates, including vitronectin, fibronectin, and laminin [39], and initiates apoptotic program via caspase-dependent and -independent mechanisms [38]. Although it was demonstrated that GrB effectively elicits apoptotic cell death in target host cells via mediating caspase activation through caspase-10, the reality may be more complex, as there are reports demonstrating that GrB may also directly activate caspase-7 or caspase-3 if the target cells lack functional caspase-10 [37,38,39]. It was also shown that GrB has the redundant capacity to initiate caspase activation, despite the absence of specific caspases. For example, it was revealed that in MCF7 cells, which express very low levels of caspase-3 and -10, a microinjection of GrB results in rapid apoptotic cell death [37,38,39]. The ability of granzyme B to induce apoptotic cell death in the presence of a partially- or completely-inactivated caspase reflects the robustness of this important cellular host defense system.

## 2. Restricted Proteolysis and Apoptotic Cell Death: Regulating Death and Preventing Inflammation-Related Danger

**Figure 2:**
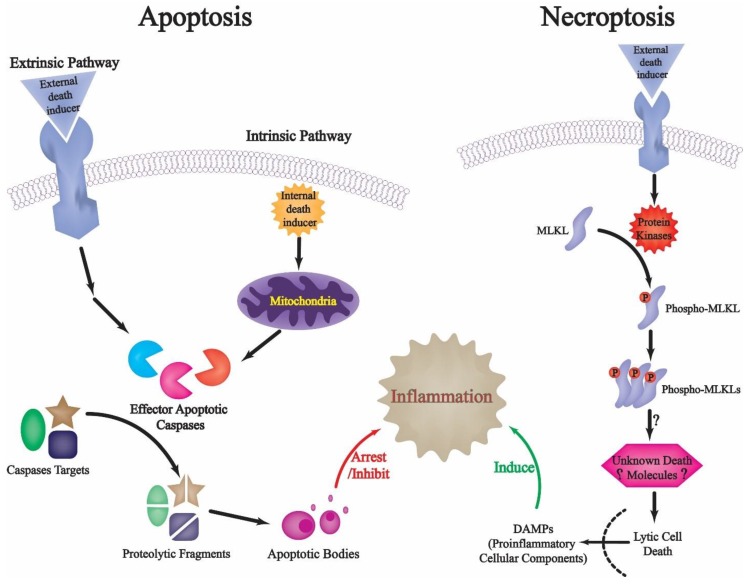
Two distinct mechanisms of cell death and impact on inflammation. In apoptosis, caspases cleave diverse signaling target substrate proteins that fine-tune the cell-death process. In necroptosis, protein kinases mediate the phosphorylation of MLKL, thereby activating it. Phospho-MLKL then leads to cell lysis that culminate in inflammatory-related roles.

Before we discuss limited proteolysis and proteases, and their roles in regulating apoptotic cell death, and how limited proteolysis can be regulated via targeted protein degradation during apoptosis, it is worth highlighting some of the crucial consequences of failing to tightly-regulate the intricate proteolytic cascades during apoptotic cell death. It has long been recognized that necrosis (that is, type III cell death) is associated with a significant loss of the integrity of the cellular plasma membrane and the subsequent release of cytoplasmic molecular cellular components into the extracellular space [22,23,26,27]. Beside the undesirable destruction to neighboring cells that such a release may provoke, there are various lines of evidence that suggest that the immune system responds to apoptotic and necrotic cells in distinctly different ways [40,41]. Necrotic cells can invariably trigger inflammation by neutrophils, macrophages, and other cells of the innate immunity, and previous work has demonstrated that this may be attributed to the release of some molecules (collectively designated as danger-associated molecular patterns (DAMPs) or alarmins) that subsequently induce pattern-recognition receptors on macrophages, dendritic cells, and natural killer cells [42]. It was revealed that stimulation of pattern-recognition receptors on innate immunity cells, particularly dendritic cells, may ultimately lead to the initiation of an immune response [43]. Thus, the presence of necrotic cells in a tissue may act as a signal to initiate immune response (Figure 2) [44].

As apoptotic cells exhibit proteases-mediated cellular signaling cascades, including plasma membrane alterations and the subsequent formation of membrane-bound apoptotic bodies which facilitate their rapid removal from tissues before rupture and release of their cytoplasmic contents [45], such cell death formats typically do not stimulate innate immune cells. Therefore, apart from limiting direct cell damage due to the release of cytoplasmic contents, one of the major benefits of tight control over apoptotic pathways, via proteolytic signaling cascades and other interconnected networks, could be to prevent the unmasking of hidden self, thereby halting unwanted immune responses. Tellingly, it has been found that alarmins such as genomic DNA and heat shock proteins are typically not released from apoptotic cells unless they appear in enormous levels that overwhelm the phagocytes capacity to dispose of them quickly [40,46]. Therefore, what happens to a cell within the apoptotic cell death program, including protease-mediated structural packaging of cellular contents and caspases-mediated alterations in the internal environment of the cell, appears to be elegantly geared towards preventing the initiation of the immune response in which the cell completes the cell death program [45].

## 3. Proteases Are Essential Elements of Apoptosis

In the early 1990s, different lines of evidence have supported a pivotal role for proteases in regulating apoptotic cell death. First, in *C. elegans*, genetic and biochemical studies revealed that apoptotic cell death is dependent on an intracellular protease (*ced-3*), bearing significant homology to the human interleukin-1b converting enzyme (ICE), which converts the 33 kDa form of IL-1b to the active 17.5 kDa form [47]. Interestingly, ectopic expression of ICE in fibroblasts leads to apoptotic cell death [48]. A second line of evidence for the crucial role of proteases in apoptotic cell death was based on the investigations that examined the role of diverse protease inhibitors on apoptosis induced by various apoptosis-inducing agents. Some of these studies have implicated calpain I, a calcium-dependent protease, in activation-induced apoptotic cell death [36,49]. Other studies that examined the the effects of CrmA, an inhibitor of ICE encoded by the cowpox virus, on apoptotic cell death further support the role of protease ICE in the induction and regulation of apoptosis. For instance, it was demonstrated that CrmA expressing Rat1 fibroblasts were protected from apoptotic death owing to serum withdrawal [50]. Moreover, a number of different intracellular proteins, including poly(ADP-ribose) polymerase (PARP) [51], lamin B1 [52], and topoisomerase I [53], have been reported to be cleaved during the onset of apoptosis, thereby supporting a direct function for the activation of one or more proteases during the process of apoptotic cell death. Subsequent reports then went on to identify 14 mammalian ICEs like protease, which are now classified as the caspases (caspase-1 to caspase-14) [54]. Although previous work has revealed that other proteases, such as cathepsins, calpains, granzymes and other non-processive proteases, may be involved in specialized and regulatory roles in apoptotic cell death [55], here, we focus caspases-dependent apoptotic cell death.

## 4. Caspases: Activation, Specificity, Function and Regulation

The core element of the apoptotic cell death machinery is a proteolytic system involving a family of proteases known as caspases. The term “caspase” dictates two pivotal features of these proteases: (i) they are cysteine proteases and exploit cysteine as the active nucleophilic moiety for cleavage of their target substrates, and (ii) they are sequence-specific, and cleave the peptide bond C-terminal to aspartic acid residues [56]. Tellingly, the notion that caspases play a pivotal role in apoptotic cell death is based on three key lines of evidence. First, inhibitors of caspases effectively halt apoptotic cell death induced by diverse apoptosis-inducing agents [57]. Second, animals lacking certain caspases exhibit prominent ablations in apoptotic cell death [58]. Third, it has been demonstrated that caspases mediate most of the identified proteolytic cleavage events that lead to the characteristic biochemical and morphological features of apoptotic cell death [11,12,13,30].

**Figure 3:**
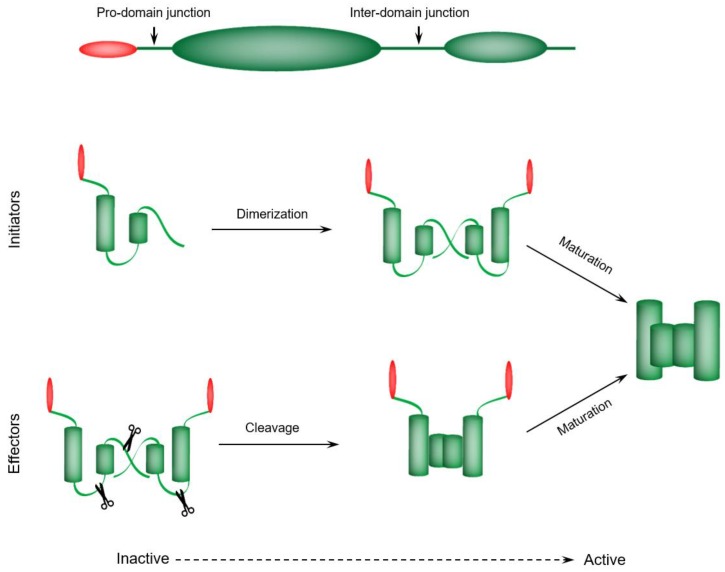
Caspases organization and activation. *Upper.* Human caspases domain organization. An N-terminal prodomain and C-terminal catalytic domain, consists of two covalently attached subunits. Locations for (auto)proteolytic events at Asp residues are labelled. *Lower.* General mechanism of activations. Initiator caspases are monomers that can be activated by pro-domain-dependent dimerization. Effector or executioner caspases are dimers that activate by the cleavage of inter-subunit junctions (linkers). Following initial activation, subsequent proteolytic events mediate the maturation process of the caspases to more stable versions.

Human caspases can be classified based on their reported function and location in cellular signaling pathways and networks. Additional criteria encompass favoring specific target substrates, and the length of pro-domain [54]. Accordingly, caspases were historically divided into apoptotic and pro-inflammatory proteases [54]. Nonetheless, most apoptotic caspases, such as caspase-2, caspase-3, caspase-6, caspase-7, caspase-8, caspase-9, and caspase-10, now have had at least one non-apoptotic biological role reported [30]. Within the apoptotic-related group, initiator caspases initiate the apoptotic signaling cascade (caspase-8, 9, 10), as described above, and their activation leads to the proteolytic activation of the executioner (or effector) caspases (caspase-3, 6, 7) [30]. As described above, initiator caspases have been further categorized into caspases participating in the intrinsic (caspase-9) or extrinsic (caspase-8 and -10) apoptotic cell death pathway [30]. Like other multi-step proteolytic cascade reactions, effector caspases are activated by limited proteolysis; yet, upstream ones, having no up steam distinct protease that mediates their cleavage and activation, respond to an activating cue via the alternative mechanisms described above [59]. Early models proposed that all caspases were activated by limited proteolysis; it is now apparent that this is only one mechanism of caspase activation. In general this holds true with the three mammalian executioner caspases (3, 6 and 7) [59]. In general, two mechanisms are involved in caspases activation, dimerization-dependent activation and proteolytic cleavage-dependent activation (Figure 3).

### 4.1. Initiator Caspases—Dimerization-Dependent Activation

In the resting state, initiator caspases are inert monomers that need homodimerization for activation [59]. In response to an apoptotic-initiating signal, caspase-dimerization occurs due to caspase recruitment to supra-molecular complex that serves as an activation platform [59]. Certain molecules derived from the supra-molecular complex (adaptors) interact with caspase recruitment domains (CARDs) of caspase-1, -2, and -9, and caspase pro-domains like death effector domains (DEDs) of caspase-8 and -10 [60]. Consequently, this recruitment and subsequent interaction mediates an augmentation in caspase levels and promotes their proteolytic activity via proximity-induced dimerization [60]. Each initiator caspase has its own supra-molecular complex or activation platform: the DISC (death inducing signaling complex) recruits and activates caspase-8 and -10, and the apoptosome activates caspase-9, while the PIDDosome may be involved in the activation of caspase-2 [54].

### 4.2. Executioner Caspases—Proteolytic Cleavage-Dependent Activation

Executioner caspases exist as inert dimers that are activated by proteolytic cleavage (Figure 3). Tellingly, these zymogens are inactive via the action of a short-peptide motif (linker or junction) that separates the large and small subunits of the catalytic domain [59]. Structural data of the zymogen form of caspase-7 revealed some of the molecular principles of the activation-induced catalytic groove formation [61,62]. Interestingly, limited proteolysis of the linker or junction permits rearrangement of some mobile loops favoring catalytic site formation [59]. It was demonstrated that, in vivo, initiator caspases (caspase-8, 9, 10) and the lymphocyte-specific serine protease Granzyme B can induce the direct activation of the downstream executioner caspases [54].

### 4.3. Caspases Maturation Events

Caspase activation is often proceeded by (auto) proteolytic cleavage events designated as maturation events (Figure 3). Tellingly, maturation events involve proteolytic excision of the pro-domain or the linker (junction) region. Notably, this activation process is essential for maturation and the subsequent induction of full enzymatic activity [59,63]. Importantly, maturation has a crucial impact at the cellular level. For instance, the caspase-8 dimer, which is not fully matured, can mediate signaling of T cell proliferation and activation yet not apoptotic cell death, which requires the fully proteolytically-processed form of caspase-8 [64]. In sum, caspase maturation is a somewhat distinct process from proteolytic activation that plays a crucial role for producing caspase metabolic stability or mediating specific downstream regulatory events.

### 4.4. Caspases Specificity

A prominent feature of each caspase’s specificity is its requirement to cleave after Asp residues [59]. Nevertheless, additional recognition elements, in most cases, need to be fulfilled to turn a polypeptide into a genuine caspase target substrate [65,66,67]. For instance, a peptide of sequence P4-P3-P2-P1-P1’, with P1-P1’ representing the scissile bond, is a genuine caspase substrate when (1) the P1 residue is Asp [66]; (2) the P1’ residue is uncharged and small (Ala, Gly, Ser) [67]; and (3) the three amino acid residues (P4-P3-P2) are complementary for interactions with the catalytic groove which promote optimal interaction and cleavage [66]. For example, it was found that executioner caspases cleave DEVD/G peptides very efficiently, yet are far less efficient with WEHD/G peptides [65].

### 4.5. Caspases Regulation

Since limited proteolysis is irreversible, activation of caspases in cells must be tightly regulated. To curb undesirable cellular responses that may result from pre-mature activation of caspases, cells exploit three counter measures to handle unscheduled caspase activation: the inhibition of activated caspases, targeted degradation of activated caspases, and decoy inhibitors [68,69,70,71]. One of the cellular strategies to inhibit activated caspases is often targeting the substrate-binding site, blocking it with a segment that mimics a genuine caspase target substrate [54,57]. One of the best characterized caspase inhibitors, CrmA (cytokine response-modifier A) from the cowpox virus, has an active site directed “suicide” inhibitors. Although it was demonstrated to mediate a rapid inhibition of caspases, this CrmA-mediated inhibition of caspases was revealed to proceed in a relatively non-specific manner [68]. Conversely, XIAP (X-linked inhibitor of apoptosis) mediates an efficient and specific inhibition of caspase-9 (*via* its BIR3 domain), caspase-3, and -7 (*via* its BIR2 domain) [72]. Tellingly, XIAP’s BIR3 and BIR2 domains mediate two specific, yet relatively weak interactions with their target caspases, to ultimately and selectively inhibit activated caspases via a two-site mechanism that is mechanistically-distinct from the viral caspase-inhibitors described above [72]. Interestingly, inhibition by decoy proteins employs proteins which are structurally related to caspase pro-domains, competing for the same adaptors within the activation platforms. Thus, they are not considered as direct inhibitors, but rather, activation preventers. For instance, FLIP (FLICE inhibitory protein), a pseudo-caspase-8 with a non-functional catalytic domain, halts caspase-8 recruitment to the DISC complex [54,68]. The final mechanism of caspase regulation involves targeted proteasomal degradation. Tellingly, activated caspases exhibited more dynamic metabolic stability profiles with respect to their inert counterparts or zymogens [73], and IAPs (Inhibitor of apoptosis-proteins) have been suggested as proteins that mediate selective degradation of activated-caspases. In addition to bearing BIR domain, many IAPs also contain RING and UbA domains that are involved in ubiquitin ligation activity [69,70]. Importantly, IAPs counteract the action of caspases through the targeted degradation of active caspases before they reach an apoptotic threshold. In line with this is the finding that mice harboring ablation in XIAP RING domain activity revealed elevated caspase activity in specific cell types, suggesting a pivotal cellular role of XIAP ubiquitin ligase activity in inhibiting activated caspases [71].

### 4.6. Does the N-End Rule Pathway Regulate Caspase-Generated Proteolytic Fragments during Apoptosis?

Following the induction of apoptotic cell death in a mammalian cell, over 1000 different proteins have been reported to be cleaved by caspases [8,9]. Although a biologically-significant subset of the resulting proteolytic fragments encompass proteins with proapoptotic (Pro-death) or anti-apoptotic (Pro-life) activity [6], there is a general lack of understanding regarding the molecular mechanisms regulating the stabilities and functions of these diverse proteolytic fragments.

As in other proteolytic activation cascades, caspase-mediated proteolytic activation of downstream signaling substrates may lead to amplification of caspase proteolytic signaling (positive feed-back loops) to promote the progression of apoptotic cell death [6,12,13,14]. In addition, proteolytic cleavage/activation of specific signaling substrates may further contribute to the biochemical and structural changes associated with apoptotic cell death in several ways: through specific structural alterations, via activation of other crucial effector signaling proteins such as nucleases (e.g., processing of pro-forms of a protein) [36,74], or via mediating the inactivation of inhibitor proteins [36].

Historically, it has been assumed that caspases activation, and the subsequent caspase-mediated-generation of proteolytic fragments during the apoptotic program, is a sign of impending cellular death. However, recent work suggested that some cells can survive beyond caspase proteolytic activation if the apoptotic cell death stimulus is ceased [75,76,77]. Moreover, previous reports have demonstrated the presence of basal activation of caspases in living cells [77]. In line with this observation, previous studies revealed that the same caspases that orchestrate apoptotic cell death programs are also implicated in diverse normal cellular roles, such as fine-tuning of neuronal activity [78], molecular mechanisms regulating learning and memory [79], and the process of spermatid individualization [80]. So, given the irreversible nature of limited proteolysis, turning off the caspase proteolytic cascades presents a distinct cellular challenge. How then, do cells evade apoptosis when unscheduled caspase activation occurs? While the activated caspases can be inhibited as described above, such as the activity of the IAPs [81], emerging evidence indicates that targeted protein degradation may also be counteracting the activity of toxic caspase-generated proteolytic products. This cellular clearance of proteolytically-activated proteins may contribute to cell survival under basal caspase activation [82].

### 4.7. How Many Protease-Generated Proteolytic Fragments Are Predicted to Be Putative Targets of the N-End Rule Degradation Pathways?

**Figure 4:**
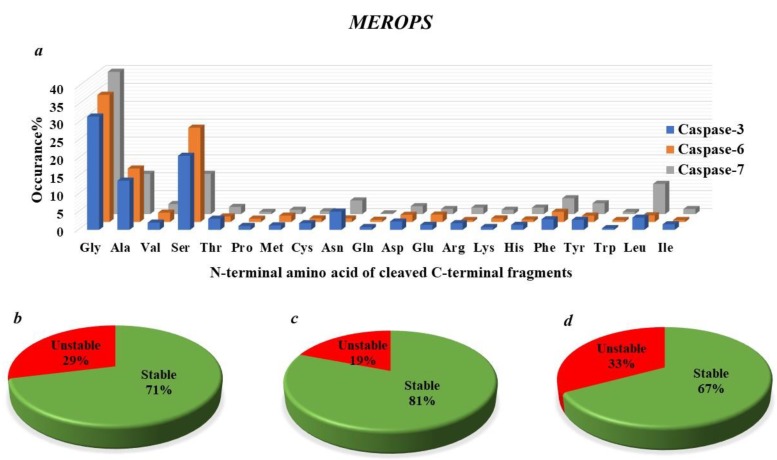
Distinct groups of proteolytic C-terminal fragments based on bearing different N-terminal amino acid residues within the database MEROPS and their occurrence % as indicated in MEROPS database (**a**); (**b**) Quantification of % of occurrence of destabilizing and stabilizing fragments generated by caspase-3 cleavage based on their theoretical half-lives; (**c**) Quantification of % of occurrence of destabilizing and stabilizing fragments generated by caspase-6 cleavage based on their theoretical half-lives; (**d**) Quantification of % of occurrence of destabilizing and stabilizing fragments generated by caspase-7 cleavage based on their theoretical half-lives.

**Figure 5:**
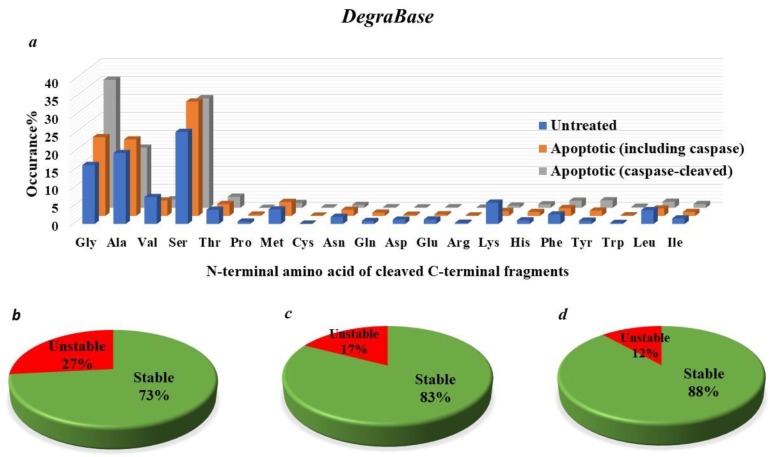
Distinct grouping of proteolytic C-terminal fragments based on bearing different N-terminal amino acid residues within the database Degrabase and their occurrence % as indicated (**a**); (**b**) Quantification of % of occurrence of destabilizing and stabilizing fragments generated by basal proteolytic processing (un-induced cells) based on their theoretical half-lives; (**c**) Quantification of % of occurrence of destabilizing and stabilizing fragments generated during apoptotic induction (including caspases cleavage) based on their theoretical half-lives; (**d**) Quantification of % of occurrence of destabilizing and stabilizing fragments generated by caspases-mediated cleavage based on their theoretical half-lives.

Previous studies have revealed that many proteolytic fragments (between 15% to 25% to of total caspase-generated proteolytic fragments) generated by caspases and archived in the databases MEROPS [83] and Degrabase [9], are short-lived (having a short half-life; less than few hours) (Figure 4 and Figure 5) [9]. Tellingly, it is estimated that over 15% of the identified caspase-generated fragments bear N-terminal destabilizing residues according to the current understanding of Arg-N-end rule degradation pathway (Figure 4 and Figure 5) [9]. Furthermore, it has been indicated, recently, that the destabilizing feature of the N-terminal residues of a subset of caspase-generated proteolytic fragments is conserved in vertebrates (the metabolic instability nature of Nt-residue is evolutionarily conserved, rather than the exact identity of the N-terminal residue of the proteolytic fragment) [10,20]. So, this evolutionarily-conserved pattern of destabilizing N-terminal residues (P1’ residue in the precursor protein) suggests that their metabolic instability is imperative for their function. Indeed, in *D. melanogaster*, it was shown that the drosophila inhibitor of apoptosis protein (DIAP) requires proteolytic priming in order to fully function as a negative regulator of executioner caspases [55,60]. Consistently, active caspases initially cleave DIAP, resulting in the exposure of a destabilizing N-terminal residue. As a result, the N-end rule degradation machinery can target DIAP for degradation via the proteasome. Thus, somewhat paradoxically, even though the N-end rule degradation pathway decreases the level of DIAP, and therefore, would be expected to reduce the apoptotic threshold, it was demonstrated that the N-end rule-mediated degradation of DIAP is important for its apoptotic function, presumably as a result of the co-degradation of the bound activated caspase.

The identity of the P1′ position of a protease cleavage site may be instrumental for the regulation of the half-life of a resulting C-terminal protein fragment, as dictated by the Arg/N-end rule degradation pathway. As discussed above, different N-terminal amino acid residues may exhibit stabilizing or destabilizing behavior, and thus, impact on the half-life of a target protein fragment. To capture a global picture of the potential for biological effects of the C-terminal proteolytic products in the context of susceptibility to the degradation via the Arg/N-end rule pathway, two proteolytic databases (MEROPS and DegraBase) were analyzed, with respect to the theoretical and predicted half-lives reported for each P1′ amino acid [9,20,83,84]. We grouped the N-terminal amino acids into stabilizing P1′ amino acid residues (half-life greater than 20 h) and destabilizing ones (half-life less than 20 h) (Figure 4b–d and Figure 5b–d). For both datasets, more than 60% of N-termini were found in the stabilizing group (half-life greater than 20 h). Tellingly, there are similarities between the frequency of stabilizing and destabilizing products depending on the proteases examined or the cellular environments or physiological states. Lastly, given that partial abrogation of Arg/N-end rule UBR-ubiquitin ligases sensitizes cells to death-inducing reagents, it would be interesting to explore caspase-independent mechanisms through which N-terminus-dependent degradation regulates cell death. This might be important not only in the cancer cell death field, but also in the context of neurodegeneration-related disorders.

These observations suggested that the Arg-N-end rule degradation pathway may play a pivotal role in curbing a significant level of caspase-mediated proteolytic generation of pro-apoptotic and anti-apoptotic fragments that bear N-destabilizing amino acid residues.

## 5. Protein Degradation by Ubiquitin Proteasome System (UPS) and the N-End Rule Degradation Pathway

In eukaryotic systems, intracellular protein degradation is mediated mainly via the action of the ubiquitin proteasome system (UPS) [20,85]. Protein degradation by the UPS system mediates the regulation of different aspects of cellular physiology, such as cellular proliferation [86], cellular division [87] cellular differentiation [88], and cell demise [10]. UPS-mediated protein degradation entails two main sequential steps: the substrate is first identified and covalently conjugated to ubiquitin via the action of highly selective E3 ubiquitin ligases. After the initial ubiquitin tagging, additional ubiquitin molecules are conjugated at lysine 48 of the ubiquitin to generate a polyubiquitin chain which is then recognized via receptors of the 26S proteasomal complex, and subsequently targeted for destruction [85,89]. Targets of the UPS pathway are ubiquitinated by a cascade of three enzymes: E1 (ubiquitin activating enzyme, using ATP); E2 (ubiquitin conjugating-enzyme or carrier enzyme) and E3 (ubiquitin protein-ligase) [85]. As UPS-mediated protein degradation is an energy-dependent and an irreversible process, tight regulation is crucial to ensure selectivity and to evade uncontrolled degradation. As such, E3 ubiquitin ligases can endow the UPS degradation network with high specificity by direct recognition of degradation signals on a target substrate [85].

The molecular elements (a region of the protein amino acid sequence and/or a conformational determinant) that confer upon protein fragments their metabolic instability are designated as primary degrons (primary degradation signals) [20,85]. Significantly, primary degradation signals can be in different forms; for instance, some primary degrons can be very simple, such as the identity N-terminal residue in the protein fragment (i.e., the N-end rule), while others may entail more complex biochemical features such as (i) PEST motif, a sequence motif that is rich in proline (P), glutamic acid (E), serine (S), and threonine (T), or (ii) the cyclin destruction box (CDB), a sequence motif that involves nine-amino acid residues, and which was originally reported in sea urchin cyclin B [90,91]. The secondary degradation signal within a protein fragment is the poly ubiquitin chain, as ubiquitin is conjugated to protein targets that bear a primary degradation signal [85]. Primary degradation signals may be buried, and hence, structurally inaccessible, or may be conditional, so that recognition requires that these signals to be initially exposed, for example via proteolytic cleavage, local unfolding, or subunit separation [92,93,94]. Another form of primary degradation signals include inducible-degradation signals, i.e., primary degrons that can be induced by the translocation from one cellular compartment to another; for example, the targeted degradation of p53, p27^Kip1^, and cyclin D when they are translocated from the nucleus to the cytosol [95,96,97].

**Figure 6:**
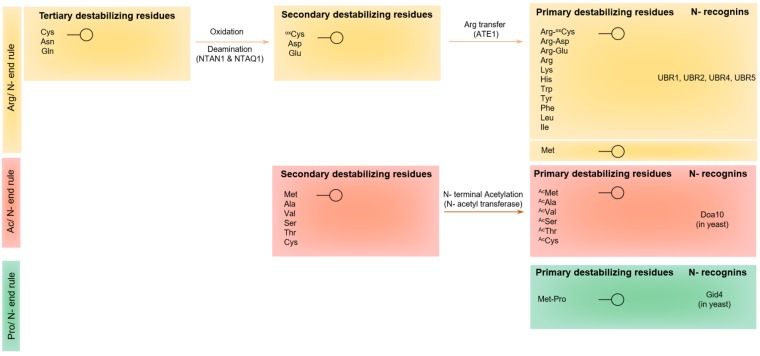
*Upper*. The mammalian Canonical N-end rule pathways. The neo-N-terminal amino acid—residue of a protein fragment may be a tertiary, secondary, or primary destabilizing amino acid residue. The conversion of tertiary and secondary destabilizing N-terminal residues are converted to primary destabilizing residues by post-translational modifications including deamidation, Cys oxidation, and arginylation. The resulting proteins are subsequently ubiquitinated and targeted to the proteasome for degradation. *Middle.* The Acetyl-N-End rule pathway. *Lower.* The Pro-N-End rule pathway.

The identity of destabilizing N-terminal amino acid residues represents the first reported degradation signals that cause metabolic instability among target protein fragments [21]. The N-end rule dictates the relationship between the half-life of a protein fragment and the identity of its N-terminal amino acid residue [20]. Different versions of the N-end rule pathway operate in prokaryotes [98], fungi [84], plants [99], and mammals [84]. The recognition components of the eukaryotic N-end rule machinery mainly entail E3 ubiquitin ligases, and they are called “N-recognins” [20]. In eukaryotic systems, the N-end rule mediated-protein degradation regulates diverse biological functions, including the elimination of abnormal misfolded protein fragments [20], the control of subunit stoichiometry (through targeted degradation) [100], the fine-tuning of chromosome repair, transcription [101], DNA damage response [102], the regulation of G protein fragments [20], the regulation of apoptotic cell death [10,103], cardiovascular development [104], peptide import [105], and other pivotal processes in plants [99]. Three branches of the N-end rue pathway operate in eukaryotes: the recently reported Pro-N-end rule pathway [106], the Ac-N-end rule pathway, which targets N-terminal-acetylated amino acid residues [107], and the classical Arg-N-end rule pathway, which targets selective unacetylated–N-terminal residues [20] (Figure 6).

The major focus of this review is the role of the Arg-N-end rule pathway with respect to caspase-generated proteolytic fragments in mammalian cell lines. In the mammalian Arg-N-end rule pathway, N-terminal amino acid residues are categorized into stabilizing, primary (1º) destabilizing (Arg, Lys, His, Leu, Phe, Tyr, Trp and Ile), secondary (2º) destabilizing (Asp and Glu), and tertiary (3º) destabilizing residues (Asn and Gln) (also Cys, under specific conditions) [18]. Proteins bearing a 1º destabilizing N-terminal amino acid residue are recognized by N-recognins (including UBR1, UBR2, UBR4 and UBR5) [108], whereas the secondary and tertiary residues are destabilizing due to their preliminary modification via Nt-deamidation (*via* NTAN or NTAQ amidases) and Nt-arginylation (*via* ATE1 transferase) [20,109] (Figure 6).

**Ac-N-End rule pathway:** Another major branch of the N-termini dependent protein degradation pathway is related to N-terminal acetylation, and has been designated as the Ac-N-End rule pathway. This specific targeted degradation pathways was shown to operate through the actions of specific E3 ubiquitin ligases that involve Doa10 in yeast cells [107] and MARCH6 in mammalian cells [107]. Importantly, since it was estimated that around 60% of the yeast proteome, and more than 90% of the mammalian proteomes, undergo N-terminal acetylation [107], this emerging signaling pathway is expected to mediate several crucial molecular processes, and significantly impact cellular proteostasis. Notably, Ac-N-end rule pathway-mediated targeting seems to be complex; this complexity may be attributed to different factors, which include additional sequence elements that can be recognized by the Ac-N-End Rule pathway, or that many of the N-termini are sterically-inaccessible to the machinery of the Ac-N-End rule degradation pathway, as this machinery was reported to degrade protein subunits that are in excess of their target complex [107].

**Pro-N-end rule pathway**: The N-terminal Proline-degron targets the gluconeogenic enzymes fructose-1, 6-bisphosphatase (Fbp1), isocitrate lyase (Icl1), malate dehydrogenase (Mdh2), and phosphoenolpyruvate carboxykinase (Pck1) for N-termini dependent degradation [106]. In this report, recent work unveiled that a Gid4 subunit, a pivotal part of GID E3 ubiquitin ligase, is the crucial factor in mediating the recognition of an N-terminal proline-containing protein substrates. A recent study by Chen et. al. [106] elucidated the selectivity of Gid4 as a versatile and specific Pro-N-recognin, and determined the molecular features that facilitate the binding of Gid4 to its substrates, including metabolic enzymes such as Fbp1 and other gluconeogenic enzymes.

### 5.1. Protein Recognition by the UBR-Box E3 Ubiquitin Ligases of the Arg-N-End Rule Pathway

In eukaryotes, a set of primary N-degrons that include positively-charged (type 1: arginine, lysine, and histidine) and bulky-hydrophobic (type 2: phenylalanine, tyrosine, tryptophan, leucine, and isoleucine) N-terminal amino acid residues can be directly recognized by N-recognins [110]. Previous work described [108] a family of mammalian E3 ubiquitin ligases that serve as N-recognins (including UBR1, UBR2, UBR4 and UBR5), characterized by a ~70-residue zinc-finger domain (amino acids 113–194) labelled as the “Ubr box domain”; in yeast, there is only one such N-recognin, Ubr1 [111]. It was demonstrated that these N-recognins bear two distinct substrate recognition domains: the Ubr box domain required for type-1 primary destabilizing residues, and the N-domain for type-2 primary destabilizing residues [20,108,110,111]. In mammals, a set of pre-N-degrons (aspartate, glutamate, asparagine, glutamine, cysteine) can destabilize proteins via N-terminal arginylation via the action of arginyl-transferase ATE1. In addition, N-terminal Asn and Gln serve as tertiary destabilizing N-terminal residues through their enzymatic deamidation via N-terminal amidohydrolases into the secondary destabilizing N-terminal residues Asp and Glu, respectively (Figure 6).

As bacteria lack the ubiquitin-proteasome system, the N-end rule degradation pathway mediates the destruction of target protein substrates via the function of ClpS, an adaptor protein that mediates the transfer of substrates to the proteolytic ClpAP complex [112]. Bulky hydrophobic (leucine, phenylalanine, tryptophan and tyrosine) and basic residues (arginine and lysine) serve as primary and secondary destabilizing residues, respectively (Figure 6). It was revealed that there are similarities between ClpS and the eukaryotic N-domain, suggesting the bacterial origin of the type-2 branch of the eukaryotic N-end rule degradation pathway [110,112]. In line with these studies, the crystal structures of ClpS [113,114] and observations from other recent work [111,112,113,114] demonstrated that the N-domain and ClpS domain adopt similar substrate-binding pockets to mediate processive protein degradation.

Structural studies of the UBR box [115,116] and of the ClpS adaptor protein (and, by homology, the N-domain of the UBR enzyme in eukaryotes) [113,114] suggest that type-1 and type-2 domains adopt different ways to bind to a variety of N-terminal amino acid residues with various sizes and forms. For instance, it was demonstrated that UBR box domains interact with type-1 peptides via a shallow and acidic groove [115,116], whereas ClpS encompasses a deep hydrophobic pocket [113] that can accommodate the binding with the hydrophobic N-terminal side chain of type-2 N-terminal amino acid residues. Tellingly, the structural data on UBR box domain show that the substrate-interacting site of the UBR box is stabilized by via a fold that encompasses two contiguous zinc fingers: a typical Cys_2_His_2_ zinc finger, and a novel binuclear zinc finger where two zinc ions in tetrahedral coordination share a common cysteine ligand [115,116].

Despite the existence of basic structural differences and a significant evolutionary gap (20), the Ubr box and ClpS show some similarity with respect to fundamental molecular principles of substrate recognition. For instance, both the UBR box and ClpS recognize the free α-amino group of the N-terminal amino acid residue via three highly-conserved hydrogen-bonds [20,112,116]. Since the N-terminus α-amino group exists in all proteins, this weak and transient binding is not substrate-selective yet can serve as a pivotal entry step in the context of the N-end rule, enabling N-recognin to rapidly scan a pool of protein N-termini. Once the N-recognin recognizes a good substrate, the interaction between the N-terminal amino acid and the UBR box domain can be stabilized via selective interactions with the N-terminus side chain [116,117], which, in turn, are further stabilized through hydrogen bonds with the first peptide bond and the side chain of the penultimate amino acid residue. Taken together. It seems that the first two N-terminal acid residues impact the recognition by the UBR E3 ubiquitin ligases. Accordingly, N-recognins appear to minimize non–N-end rule specific interactions with the rest of the protein by limiting the major interactions to the first two N-terminal amino acids. This two-step N-end rule recognition provides the molecular basis for binding selectivity and affinity between the Ubr box and a peptide substrate [116,117].

As the N-recognin binding to the target N-terminal amino acid proceeds, the N-recognin E3 ubiquitin activity mediates E2-dependent conjugation of ubiquitin to a properly-exposed lysine residue on the target substrate [116,117]. Tellingly, significant evidence suggests that a genuine N-end rule substrate may encompasses an unstructured, flexible region between the N-terminal amino acid residue and the rest of protein fragment [116,117], which may be needed to search for a polyubiquitination site. Once ubiquitin chain begins to assemble, the N-recognin can be released from the substrate N-terminal residue to initiate a new round of N-end rule recognition. It is noteworthy that recent biochemical studies suggest that UBR box domains have relatively high dissociation rates, revealing the reason behind the high processivity of the N-end rule-mediated degradation found in eukaryotic cells [21,84,117,118].

Although structural work reveals the crucial importance for the identity of the N-terminal amino acid residue, mounting evidence suggests that the second amino acid residue also significantly contributes to N-end rule recognition and selectivity; yet, much is still unknown regarding a unifying principle for the importance of the second residue [109]. Previous structural work demonstrated that the human Ubr box domain favors acidic amino acid residues in the penultimate position, which exist in the products of N-terminal arginylation of secondary N-terminal destabilizing residues [116]. Remarkably, bacterial ClpS also prefers arginine or lysine at the second position [112]. It thus seems that the second N-terminal amino acid residue significantly impacts the affinity to the Ubr box in a strategy which is specific to individual N-recognins. Indeed, more recent work has shown that the mouse version of Ubr1 and Ubr2 can recognize proteins having the unacetylated N-terminal Methionine if it is followed by bulky hydrophobic amino acid residues (as Leu, Ile, Tyr, and Trp). Tellingly, ubiquitination data and binding assays suggested that this N-degron can be recognized by the type II binding site of UBR1 or UBR2 [119].

### 5.2. The N-End-Rule Pathway vis-à-vis Caspase-Generated Proteolytic Fragments: The Beginning Determines the End

Upon the activation of the proteolytic cascades in the context of apoptotic cell death, an enormous number of various proteolytic cleavage events take place. Tellingly, many of these cleavage events culminate in activation, inactivation, or alteration in cleaved proteins functions and localization. This proteolytic reprogramming of a protein can facilitates sculpting the apoptotic program, and is exemplified by the cleavage of crucial signaling kinases such as RIPK1, BMX kinase, and PKC-δ.

In contrast to the current model about the counteracting responses to activated caspases [7], our understanding about the counteracting molecular responses to caspases-generated proteolytic fragments (pro-apoptotic and anti-apoptotic fragments) is still far from clear [20]. It has recently been revealed that the destabilizing feature of the N-terminal residues of a cellular-significant subset of caspase-generated proteolytic fragments is fully conserved in vertebrates (the metabolic instability nature of Nt-residue is conserved, rather than the exact identity of the N-terminal residue of the proteolytic fragment) [9,10,20]. This evolutionarily-conserved pattern of destabilizing N-terminal residues (P1’ residue in the precursor protein) suggests that their metabolic instability is imperative for their functional attributes. Accordingly, it is possible that the N-end rule degradation pathway acts as one of the molecular mechanisms that counteracts these caspase-generated proteolytic fragments (either pro- and anti-apoptotic fragments) via targeted protein degradation that may be generated by basal and induced protease activity [10].

## 6. The N-End Rule Pathway and Regulation of Apoptosis

The elucidation of the functions and roles of the Arg-N-end rule degradation machinery with respect to apoptotic cell death is confounded, at least in part, by the strikingly diverse potential N-end rule target protein substrates in the mammalian cellular system and the complexities of apoptotic cell death signaling cascades during different apoptotic phases (initiation and execution phases) [9,10,20,103,109,120,121]. Previous work demonstrated that several proteolytic protein fragments (caspase activated fragments of Lyn kinase, BMX kinase, BRCA1, RIPK1, and several others) are short-lived substrates of the Arg/N-end rule pathway in mammalian-derived cell lines. The entire set of caspases-generated proteolytic fragments that are potentially short-lived Arg/N-end rule substrates certainly comprises more than the currently-reported substrates (Figure 4 and Figure 5) [9], and several other previously-identified proteolytic fragments remain to be examined for their degradation via the N-end rule degradation machinery. Furthermore, more of such active proteolytic fragments are likely to be identified in future studies (Figure 4 and Figure 5). By targeting these fragments for proteasomal degradation, the Arg/N-end rule pathway counteracts their caspases-mediated activation, and thereby, dampens their pro-apoptotic or anti-apoptotic reactions that regulate apoptotic signaling [121].

## 7. The N-End Rule Degradation Machinery Counteracts Pro-Apoptotic Cell Death Signaling

**Figure 7:**
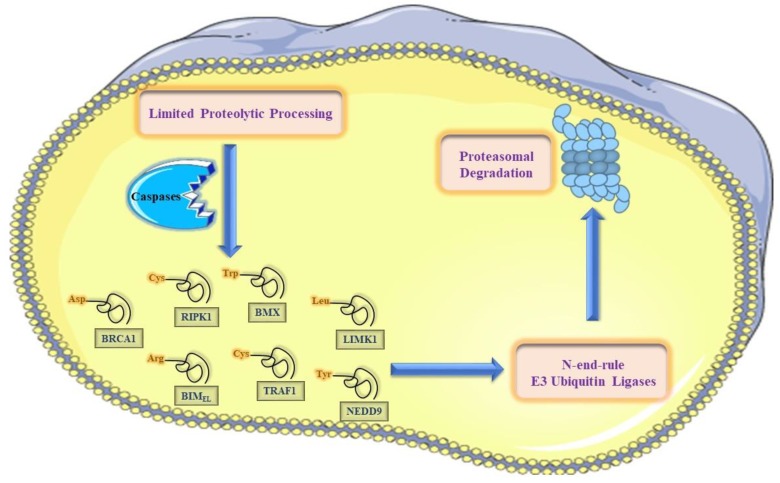
Schematic depiction of the anti-apoptotic role of the Arg/N-end rule pathway via targeted degradation of several pro-apoptotic protein fragments during induction of apoptosis in the context of apoptotic cell death signaling. In addition to other antiapoptotic pathways, the selective degradation of proapoptotic protein fragments via the Arg/N-end rule pathway curbs transient or otherwise unscheduled apoptotic signaling from reaching the point of cellular demise. All the indicated pro-apoptotic fragments have been described in detail in references 10 and 120.

Protease-generated proapoptotic fragments likely act as components of positive feedback loops that amplify specific aspects of apoptotic cell death, including caspase activation [10]. Tellingly, several kinases exhibit elevated kinase activity once their N-terminal regulatory domain is removed by proteolytic cleavage events as part of positive amplification loop during the context of apoptosis [122,123,124]. One example of such protein kinase fragments is the C-terminal fragment of RIPK1. Previous work revealed that the pro-apoptotic cleaved fragment of RIPK1 ΔN is unstable in T-Rex human-derived cells, as its N-terminal cysteine targets it for proteasomal degradation via the Arg-N-end rule pathway, and this degradation is inhibited by mutating the destabilizing N-Termini into the otherwise-stable N-terminal valine. Significantly, it was demonstrated that the metabolic stabilization of the cleaved fragment of RIPK1 or the inhibition of the N-end rule machinery augments the apoptosis-inducing effect of diverse apoptosis-inducing agents in in human T-Rex-293–based cell lines. Concomitantly, our work on the cleaved form of BMX kinase has further extended this N-end rule anti-apoptotic function in other human-derived cancer cell lines (Figure 7) [120].

We also demonstrated, for the first time, that the caspase cleaved form of the BMX tyrosine kinase is actively degraded by the N-end Rule pathway in prostate-derived cancer cell lines. Interestingly, we have also discovered, that the N-end rule-mediated degradation of the cleaved pro-apoptotic BMX kinase dampens its pro-apoptotic function. Collectively, our data on the emerging role of the N-end-Rule pathway in attenuating the apoptotic program via destroying active pro-apoptotic kinase fragments have revealed this pathway to be a potential target to sensitize cancer cells to cell-death promoting therapies.

## 8. The N-End Rule Pathway Can Mediate Pro-Apoptotic Signaling

Intriguingly, our previous work revealed that the N-end rule pathway can target the anti-apoptotic LynΔN for proteasomal-degradation, and thus, dampens its anti-apoptotic function, suggesting that the N-end rule degradation machinery can mediate a pro-apoptotic role. The proteasomal-mediated degradation of LynΔN is a result of the proteolytically-exposed N-terminal leucine being recognized by the N-end rule degradation machinery. Tellingly, we have demonstrated that degradation can be prevented by changing the identity of the N-terminal amino acid to valine. The complete conservation of N-terminal leucine in vertebrates suggests the importance of this destabilizing residue as a major functional attribute of this active anti-apoptotic kinase fragment.

The degradation of LynΔN by the N-end rule pathway provides an example of how the N-End rule degradation pathway can function in a pro-apoptotic manner. This was demonstrated by how the valine-LynΔN stabilizing mutant provides significantly higher imatinib resistance to K562 cells. A previous example of N-End rule-dependent cell death has been previously described with macrophages treated with anthrax lethal toxin [125]. In addition, recent work revealed that the Arg-N-end rule pathway enzyme Ate1 can promote cell death and/or growth arrest, depending on the context, type, and level of stress stimulus [126]. However, the detailed molecular basis for these observations are far from clear, and molecular mechanistic studies in disease-relevant cellular models are warranted to extend our physiological understanding with respect to the previous observations.

## 9. The N-End Rule-Mediated Degradation of a Caspase-Generated Fragment Can Be Modulated by Phosphorylation

Although some pro-apoptotic fragments have been validated as short-lived substrates for the N-end rule pathway, we have found that the N-end rule-mediated degradation of the cleaved BMX kinase is inhibited by its phosphorylation [120]. This is an entirely novel mechanism of regulation, as phosphorylation of a protease-generated substrate has never been reported to alter the N-End-Rule degradation pathway, which was discovered three decades ago [21]. Our work also addresses some of the duality reported regarding the pro/anti-survival function of the BMX kinase [127,128]. Overall our findings have revealed an unforeseen interplay between the phosphorylation and N-end-Rule-protein degradation of a caspase product, revealing an increasingly-complex regulatory network of apoptotic signaling cascades.

It is yet to be explored if there are other N-end rule substrates that can be regulated by phosphorylation. Although there are a number of examples of how phosphorylation impacts, either positively or negatively, target protein substrate degradation by the ubiquitin-proteasome system [129], our work presented for the first time a clear example of phosphorylation regulating recognition of a caspase proteolytic product via the N-end rule degradation pathway, which was discovered about 3 decades ago. The mechanism of how internal phosphorylation inhibits recognition and proteasome targeting by UBR1 and UBR2 has yet to be explored.

## 10. Mutual Repression between the N-End Rule Pathway and Apoptotic Activated Proteolytic Machinery

Given the significant counteracting response of the Arg/N-end rule pathway to caspase-generated pro-apoptotic fragments [10,109,120,130], might there be a mutual repression between this degradation machinery and critical proapoptotic effectors such as activated caspases? Recent work demonstrated that key recognition components of the Arg-N-end rule degradation machinery are abrogated upon apoptotic cell death progression [10]. Tellingly, it was found that ATE1 arginyl-transferase is degraded in apoptotic cells, that activated caspases can mediate the cleavage of ATE1 arginyl-transferase and the UBR1 E3 Ubiquitin ligase, and that these cleavage events can functionally inactivate ATE1 arginyl-transferase, indicating a dynamic inhibitory cross-talk between the Arg/N-end rule degradation machinery and the potent proapoptotic activated caspases [10]. A tempting interpretation for this mutual repression dictates that the N-end rule degradation of caspase-generated proteolytic fragments would be relatively high at the early stages of apoptotic cell death signaling [10]. However, upon progression of apoptotic cell death signaling, caspase-mediated suppression of the N-end rule degradation machinery would increase, specifically at later apoptotic phases, when a potent proapoptotic signaling begins to outplay the antiapoptotic response of the Arg/N-end rule pathway and other antiapoptotic networks in a manner that the proapoptotic signaling reaches the point of cellular demise or the point of no return [10].

## 11. Concluding Remarks and Future Directions

### 11.1. The N-End Rule Degradation Pathways and the Non-Apoptotic Functions of Caspases: Is There A Link?

It is well known that effector caspases mediate cellular shrinkage, membrane blebbing, and disintegrating into distinct apoptotic bodies which collectively result in apoptotic cellular demise [22,36,131]. Moreover, emerging lines of evidence are revealing that effector caspases also mediate other non-apoptotic cellular roles in developmental signaling, cellular homeostasis, and post-injury signaling cascades [132,133,134]. The activation of effector caspases have been demonstrated during spermatogenesis [135], lens development [136], stem cell differentiation [137], erythroid differentiation [138], neuronal cellular development, and dendrite pruning [139,140], cell migration [141], and cell shaping [142].

In addition to the emerging roles for caspases during normal healthy processes, alternative non-apoptotic functions are being identified in cancers. This is exemplified by the observations that sub-lethal activation of caspases may culminate in cellular differentiation and the epigenetic reprogramming of cells in a tumor [143], and that the spontaneous, uninduced activation of caspases in many cancer cells plays an instrumental function in maintaining their tumorigenicity and metastatic behavior [143]. In addition, a recent report has suggested that caspase 3 can regulate non-apoptotic functions in colon cancer cell lines such as migration, invasion, and metastatic potential [133].

An intriguing aspect of the apoptotic program in cancer cells pertains to the reversibility of apoptosis (Anastasis) in some cancer cells [144,145]. While several types of cancer cells undergo noticeable initial responses to cell death-inducing agents such as chemotherapeutic or radiotherapeutic approaches, these cells may nonetheless relapse even after reaching the assumed “point of no return”, such as by the MOMP-mediated activation of effector caspases such as caspase-3 activation, and ultimately, metastasis often takes place in many types of cancer [144]. Interestingly, recent work has revealed that cancer cells recovering from apoptotic cellular demise exhibit more aggressive behavior such as having higher tumorigenic potential and metastatic potential in vivo, and that the reversal of apoptotic cell death may elicit the generation of new CSCs from NSCCs in breast cancer cells [145]. While some emerging functional implications have been reported for the reversal of apoptosis in cancer cells [144,145], the details of molecular and biochemical mechanisms regulating caspases activity during this process are yet to be discovered. Future work needs to address the biochemical mechanisms, including the role of proteasomal degradation pathways such as the N-end rule degradation pathways, that can potentially fine-tune the activity of caspases during the reversal of apoptosis.

Given the cellular role of the N-end rule degradation pathway in the turnover of caspase-activated fragments, it would be interesting for future studies to investigate the role of N-end rule enzymes in regulating cancerous cell behavior such as invasion, migration, and metastasis. In line with this, recent investigations on clinically-derived tumor arrays have suggested that Ate1, an enzyme of the Arg/N-end rule pathway, may be down-regulated in some human cancers, such as kidney and colon, at the protein level. Further analysis on larger clinical datasets revealed that the expression of Ate1 mRNA in some of these tumors is inversely correlated with the metastatic stage of the disease and patient survival [146].

An important aspect remaining to be examined regarding the emerging alternative roles for caspases is their regulation. How do some cells transiently activate some caspases to carry out their function in a controlled manner, and then ultimately deactivate them while preventing the activation of apoptosis [147]. Endogenous mechanisms for counteracting the activity of caspases are known, such as the direct interaction of caspases to members of the inhibitor of apoptosis protein (IAPs) family that include the E3 ubiquitin ligases [148]. Alternative routes exist, and include the action of some E3 ubiquitin ligases that may recruit caspases, and subsequently target caspases for proteasomal-dependent degradation [149,150]. In this context, the proteasomal-dependent N-end rule-mediated protein degradation can emerge as an additional pivotal regulatory control layer within the operation of caspases in non-apoptotic functions. Indeed, this is exemplified in a recent report describing the caspase CED-3 (an orthologue of caspase-9) cleavage of the LIN-28 protein to limit its activity during seam-cell patterning and differentiation in *C*. *elegans* [151]. This work reveals that this non-apoptotic function of CED-3 proceeds with the recruitment of the UBR1 ubiquitin ligase, and that the functional operation of both proteins is necessary for the proper recognition and subsequent degradation of LIN-28 by the proteasome. This work revealed a functional coupling between CED-3 and the Arg/N-end rule UBR1 E3 ubiquitin ligase in the context of a gene expression program that fine-tunes temporal cell fate patterning of seam cells in *C*. *elegans*. Furthermore, the evidence that the CED-3 caspase and UBR-1 can interact and form a complex that rapidly targets the LIN-28 protein for degradation in vivo underscores the role of N-end rule degradation in assisting caspases in dynamically shifting stage-specific developmental programs. Interestingly, the requirement of the UBR1 E3 ubiquitin ligase by CED-3 caspase to initiate LIN-28-targeted degradation points to a molecular model for differential recognition of a non-apoptotic protein fragment [151]. Future investigations may reveal similar associations between the UBR ligases and caspases, and will require detailed molecular and biological investigations, such as those conducted on gasdermin and ICAD [147,152].

### 11.2. Beyond the N-End Rule: The N-End-Code beyond the Mere Identity of the N-Terminal Amino Acid Residue

The N-end rule pathway has been postulated as a rule that dictates the relationship between the nature of the N-terminal amino acid residue and the half-life of a given protein. Mounting lines of evidences show that the actual disposition is much more complex than is currently appreciated [106,107]. For instance, N-terminal acetylation of the N-terminal amino residue has been demonstrated to trigger protein degradation via creating specialized degrons called (Ac-N-degron) that can be recognized by the so-called Ac-N-end rule pathway [107]. Notably, it has been demonstrated recently that the generation of Ac/N-degrons contributes to protein quality control networks and the fine-tuning of intracellular protein homeostasis [100].

Another emerging pivotal factor in the regulation of the N-terminal-dependent protein degradation is the identity of second amino acid residue. Recent lines of evidence support a crucial role for the identity of amino acid residue in the penultimate position in recognition and subsequent degradation via specialized N-end rule degradation components [106]. For example, recent work in yeast demonstrated that unacetylated N-terminal methionine can trigger the degradation of a given protein if the second amino acid residue is bulky hydrophobic residue, and that this degradation depends on the yeast UBR1 N-recognin [119]. Furthermore, more recent work has shown that Pck1, a gluconeogenic enzyme found in yeast which contains Pro at position 2, can be targeted for degradation by a new branch of the N-end rule pathway called “Pro-N-end rule pathway”. Gid4 was identified as the N-recognin of the Pro-N-end rule pathway. While the structural basis of this work is far from clear, this work revealed that recognition depends on the identity of the first four N-terminal amino acid residues [106]. It is tempting to speculate that future work may unfold some of the complexity of N-degron, which could encompass a combination of features, like the identity of N-terminal residue, specific PTM, specific structural features, and certain localizations features [153,154].

Recent work has revealed that the N-terminal dependent protein degradation (the Arg-N-end rule pathway and other branches of the N-end rule) may potentially target significant number of caspase-generated C-terminal proteolytic fragments (estimated to be around 20 to 30% of total caspases-generated protein fragments according to MEROPS database, and 15 to 25% of total cleaved proteolytic fragments according to Degrabase database) (Figure 4 and Figure 5) [9,83]. Nonetheless, many of these proteolytic products have other N termini that are not recognized by the N-end rule-proteasomal degradation machinery, and may be targeted by other degradation machineries [9,83]. In addition, there are also some functional N-terminal proteolytic fragments of the protein substrates to be considered as well [20,155]. It is likely that N-end rule degradation machinery acts as one of the components of the signaling networks occurring during these proteolytically-active cascades.

In the context of the pivotal role of ATE1-mediated N-terminal arginylation in mediating the recognition of some C-terminal proteolytic fragments bearing specific N-terminal-destabilizing residues by the Arg/N-end rule pathway, it is tempting to speculate about whether there might be a hypothetical activity that could counteract arginylation in vivo (de-arginylation enzyme(s)), which may be capable of removing post-translationally-added Arg from substrate protein fragments in an analogy to ubiquitylation and deubiquitylation and their role in cellular physiology [156]. Examining the question of whether these or other similar enzymes may be involved in regulation of arginylation in vivo provides an interesting route for future research regarding fine-tuning the metabolic stability of active proteolytic fragments during the context of apoptosis signaling [154].

Given the pivotal role of non-processive proteases and cysteine oxidases in the production of N-end rule proteolytic substrates during apoptotic signaling, work aiming at defining target substrates for N-end rule degradation pathways would be greatly strengthened by exploiting novel proteomics state-of-the-art techniques, designated as N-terminomics, to help in identifying and characterizing neo-N-terminal amino acid residues within target substrates and their PTMs at the global cellular level (e.g., oxidation, arginylation).

### 11.3. Might the Arg/N-End Rule Pathway Serve as A Modulator of Apoptosis?

Recent work on identifying several pro-apoptotic protein fragments as target substrates for the Arg/N-end rule pathway might open up future avenues of research regarding utilizing N-end rule pathway recognition components as a versatile modulator of apoptosis in clinically- and physiologically-relevant cellular conditions via the specific alteration of the activity and/or abundance of key components of the Arg/N-end rule pathway. The possibility of elevating or reducing specific parts of the Arg/N-end rule pathway opens up pharmaceutical opportunities to selectively alter the output of apoptotic cell death circuits. However, some further questions still need to be addressed to enhance our molecular and physiological understanding regarding the role of the N-end rule pathways during apoptotic cell death. A crucial question pertains to whether the N-end rule components are selectively regulated (upregulation or downregulation) during specific physiological inputs or during different signaling periods. Some recent data, in cultured cells and in vitro, about the cleavage of ATE1 and UBR1 by caspases support this conjecture. However, further studies using additional, more physiologically-relevant models would be warranted to dissect this question. Interestingly, recent work on flies has shown that that viral infection elicits an elevation of caspase-generated *Drosophila* inhibitor of apoptosis 1 (DIAP1), a target substrate of the Arg/N-end rule in flies, by invoking the targeted proteasomal-degradation of N-terminal amidohydrolase 1 (NTAN1) [157].

Given the striking range of diversity of predicted proteolytic protein substrates of N-end rule pathways within the context of apoptotic signaling, it is likely that the N-end rule pathways-mediated regulation of cell death entails additional processes or molecular links. In line with this, given the recent developments in methods to study protein-protein interactions (mass-spectrometry-based methods or AP-based methods coupled to Mass-spectrometry), it is expected that future studies may identify novel N-end rule degradation components and novel N-end rule target substrates that are relevant to distinct cellular physiological inputs, and further unfold the role of N-terminal-dependent-protein turnover on the cellular biology of cell death with potentially far-reaching implications.
